# Retraction: From molecular mechanism to plant intervention: the bidirectional regulation of inflammation and oxidative stress in bone aging

**DOI:** 10.3389/fendo.2026.1792387

**Published:** 2026-01-29

**Authors:** Frontiers Editorial Office

The Journal retracts the 2025 article cited above.

Following publication, the publisher has found evidence that the peer review process was compromised. As the scientific integrity of the article cannot be guaranteed and adhering to the recommendations of the Committee on Publication Ethics (COPE), the publisher therefore retracts the article.

Please note that we have not confirmed whether all or any of the authors were aware of or involved in any form of misconduct or manipulation of the publication process.

This retraction was approved by the Chief Editors of Frontiers in Medicine and the Chief Executive Editor of Frontiers. The authors received a communication regarding the retraction and had a chance to respond. This communication has been recorded by the publisher.

